# Supplementary material for: GATA3 zinc finger 2 mutations reprogram the breast cancer transcriptional network
Source: Nat Commun. 2018 Mar 13;9:1059. doi: 10.1038/s41467-018-03478-4 (PMC5849768; doi:10.1038/s41467-018-03478-4)
Supplement: Supplementary file 1 — Supplementary Information [file 41467_2018_3478_MOESM1_ESM.pdf]

GATA3 Zinc Finger 2 mutations reprogram the breast cancer transcriptional network

Takaku et al.

Takaku et al. Supplementary Figure 1

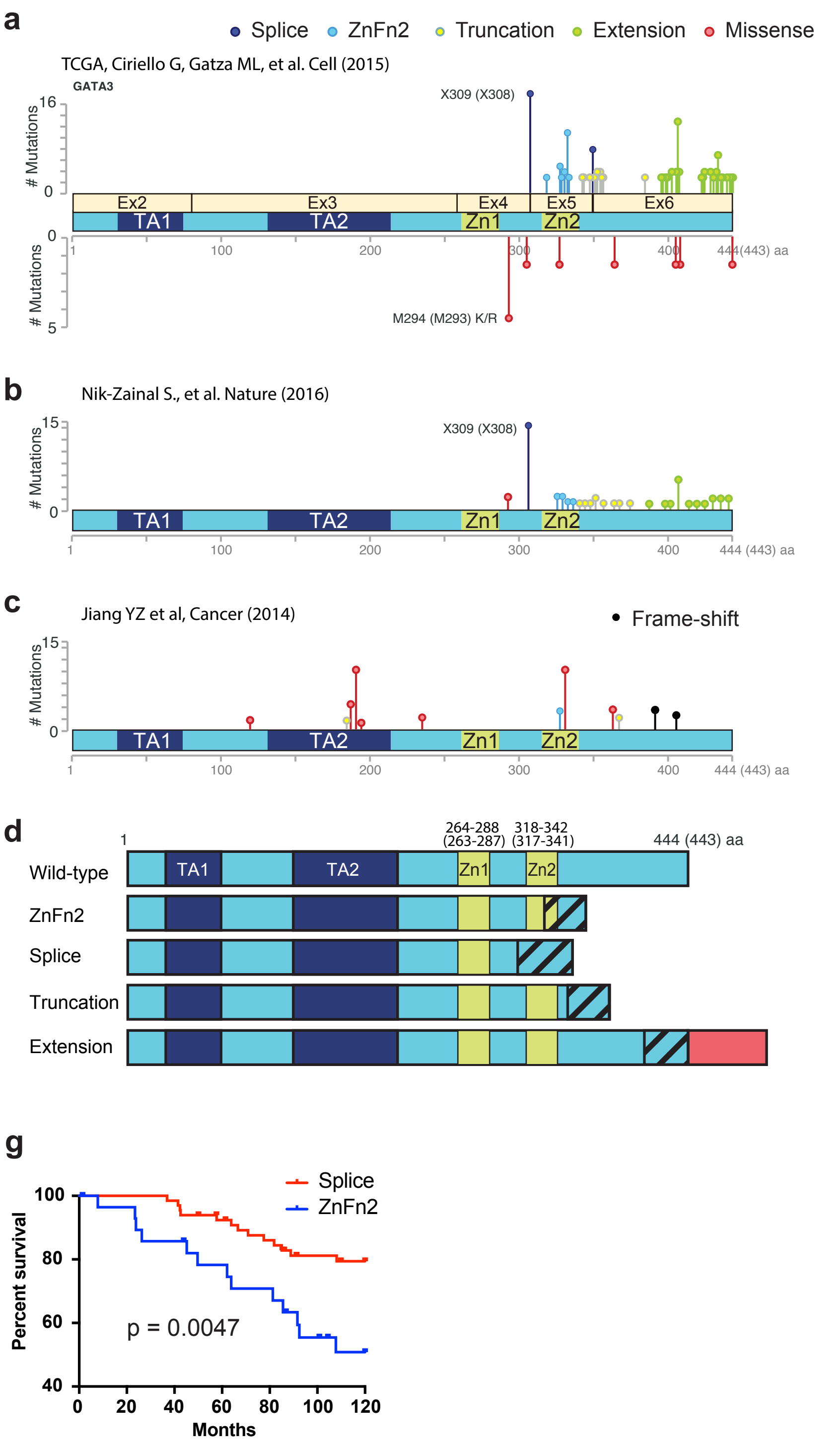

e

Luminal A and B frequency (Fisher's exact test)

METABRIC

	GATA3 WT	Splice	Truncation
ZnFn2	0.0254	<0.0001	0.0026
Splice	0.0002	-	0.7664
Truncation	0.0397	-	-

TCGA

	GATA3 WT	Splice	Truncation
ZnFn2	0.0121	0.0062	0.1493
Splice	0.215	-	0.4007
Truncation	>0.9999	-	-

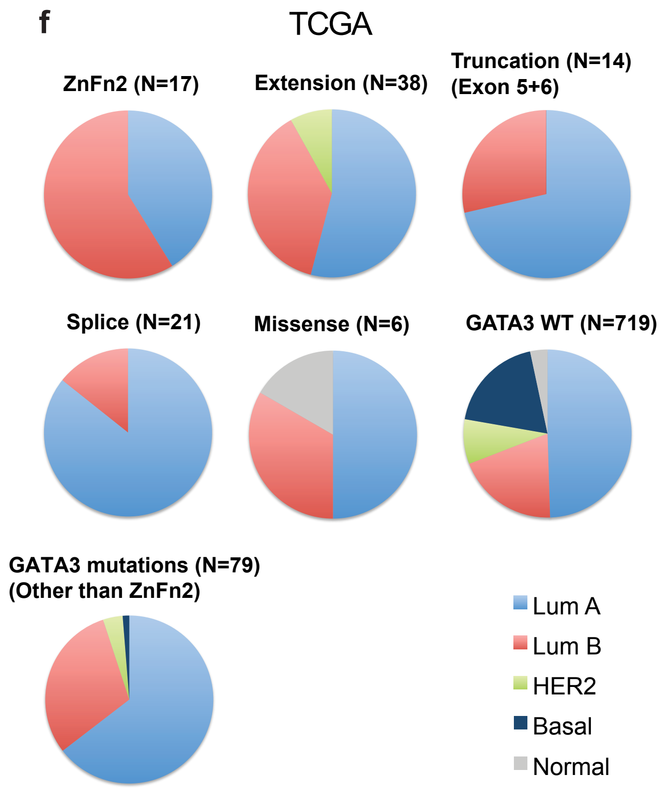

Supplementary Figure 1. Distribution of GATA3 mutations in breast tumors.
(**a**) GATA3 mutations found in the TCGA data cohort (Ciriello et al., 2015; The Cancer Genome Atlas Network, 2012). TA1 and TA2 (dark blue) indicate transactivation domains. Zinc finger motifs ('Zn1' and 'Zn2') are depicted in green. (**b**) GATA3 mutations taken from Nik-Zainal S. et al (Nik-Zainal et al., 2016). (**c**) Distribution of GATA3 mutations identified by Jiang Y.Z. et al. (Jiang et al., 2014). (**d**) Representative protein structures of GATA3 mutants. The hatched region in the mutant indicates missense amino acids. The extension regions are shown in red. The ZnFn2 class is defined as any type of mutations found between 318 and 342 amino acids residues. Typical ZnFn2 mutations are frame-shift mutations, resulting in protein truncation with ~20 missense amino acid residues. The splice mutations induce alternative splicing and frequently observed at the exon 4/5 junction. These mutations also produce truncated version of the GATA3 protein with ~40 missense amino acid residues. The other truncation mutations are frequently found in Exon5 and Exon6. The extension mutations are frame-shift mutations, which alter the reading frame of the C-terminal region to the same reading frame. This results in protein extension (~60 amino acid residues). (**e**) ZnFn2 tumors are freqeuntly observed in luminal B tumors. Fisher's exact test was performed to test statistical differences of luminal A and B frequencies between each GATA3 mutation group. P-values of each comparison are indicated (Left: METABRIC data cohort, Right: TCGA data cohort). For the METABRIC data analysis, high GATA3 expression cases (defined in Fig. 1a) were used. (**f**) Breast cancer subtype distribution in GATA3 mutant tumors. The PAM50 classified subtype information was collected from the TCGA data cohort. (**g**) Kaplan-Meier survival curves from different classes of GATA3 mutations. Luminal A and B tumor cases were used to generate the survival curve. Log rank p-values are indicated.

Takaku et al. Supplementary Figure 2

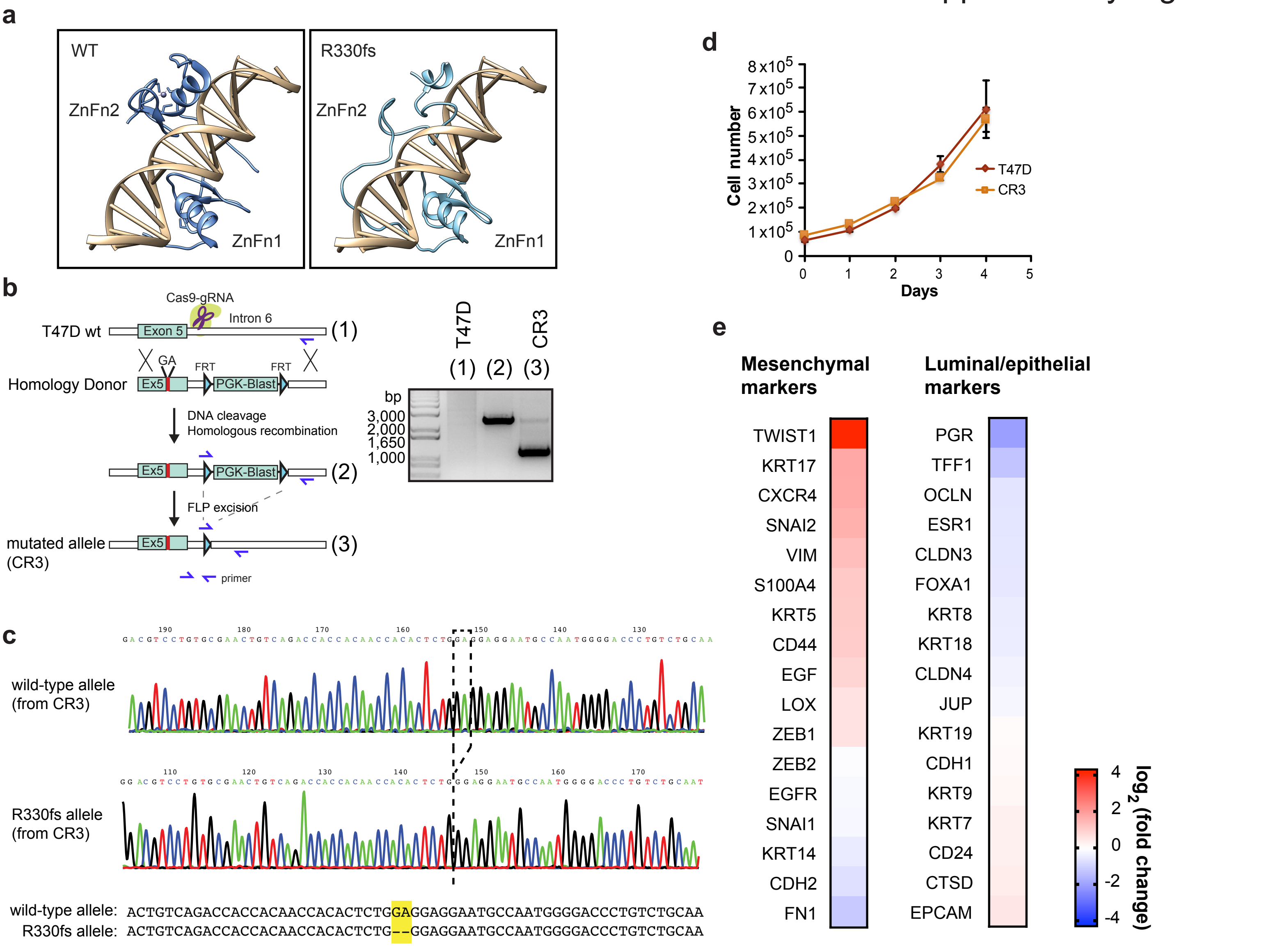

f

Pathway analysis of Down-regulated genes

Molecular and Cellular Functions	p-Value	Activation z-score	Molecules
Generation of cells	1.38E-06	-3.788	32
**Development of neurons**	5.71E-05	-2.557	16
Morphogenesis of neurites	7.01E-05	-2.785	11
**Differentiation of cells**	9.64E-05	-2.718	30
Formation of plasma membrane projections	1.36E-04	-2.538	13
Fertility	2.90E-04	-2.345	9
Neuritogenesis	3.76E-04	-2.341	12
Cellular homeostasis	5.45E-04	-3.269	22
Formation of cellular protrusions	8.24E-04	-2.741	15
Size of body	1.03E-03	-3.091	14
Microtubule dynamics	1.80E-03	-2.652	17
**Differentiation of mononuclear leukocytes**	2.00E-03	-2.571	10
Shape change of neurites	2.08E-03	-2.6	7
**Differentiation of leukocytes**	3.44E-03	-2.225	11
**Differentiation of lymphocytes**	3.61E-03	-2.382	9
Cell movement of leukocytes	3.63E-03	-2.012	12
Quantity of cells	5.01E-03	-2.721	21
**T cell development**	5.62E-03	-2.759	9
Quantity of lymphoid tissue	6.81E-03	-2.318	7
Branching of neurites	7.94E-03	-2.4	6

g

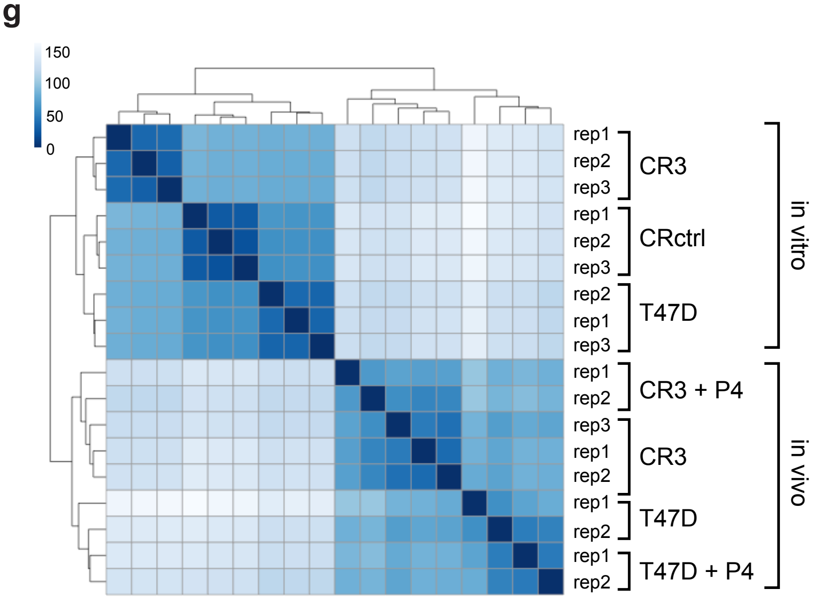

Supplementary Figure 2. Gene expression profiling of CR3 R330fs mutant cells.
(**a**) Model structure of the DNA-binding domain (DBD) of the R330fs mutant. The wild-type DBD structure was utilized to generate the model structure. The wild-type and mutant DBDs are presented in cyan and light blue respectively. (**b**) PCR validation of the R330fs CRISPR clone. The homology dsDNA template (which contains R330fs mutation) was co-transfected with the Cas9-gRNA expression plasmid. After Blasticidin selection and single cell cloning, CR3 positive cell clone was selected by the PCR analysis. The blasticidin cassette was removed by FLP recombinase. The blue arrows indicate primers used for the PCR amplification (right panel). (**c**) Sequence confirmation of the CR3 mutant cells. The two-nucleotide deletion in the CR3 cells is highlighted. GATA3 Exon 5 region was amplified from the CR3 cells, and the PCR product was cloned into the pCR4-TOPO TA vector for sanger sequencing. 3 out of 10 clones contained 'GA' nucleotide deletion. (**d**) Cell proliferation analysis of CR3 cells. T47D control cells and CR3 cells were grown in high glucose DMEM medium containing 5% FBS. Mean cell numbers were plotted with SD (N=3). (**e**) Heatmap showing the expression levels of mesenchaymal marker genes, luminal and epithelial marker genes. Differential gene expression between control and CR3 cells (obtained from DESeq2) was indicated. (**f**) The top molecular and cellular function related pathways. The IPA analysis was conducted by using top 100 down-regulated genes (ranked by fold-changes). The top 20 bio function pathways (ranked by p-values) are indicated. (**g**) Clustered heatmap showing expression patterns in T47D cells , CR3 cells, and control CRISPR-Cas9 clone (CRctrl) cells. The scale indicates Euclidean distance. 'in vitro' indicates the RNA-seq data from in vitro cell culture system, while 'in vivo' indicates the RNA-seq data from mouse xenograft model. P4 indicates xenograft experiments with progesterone pellets.

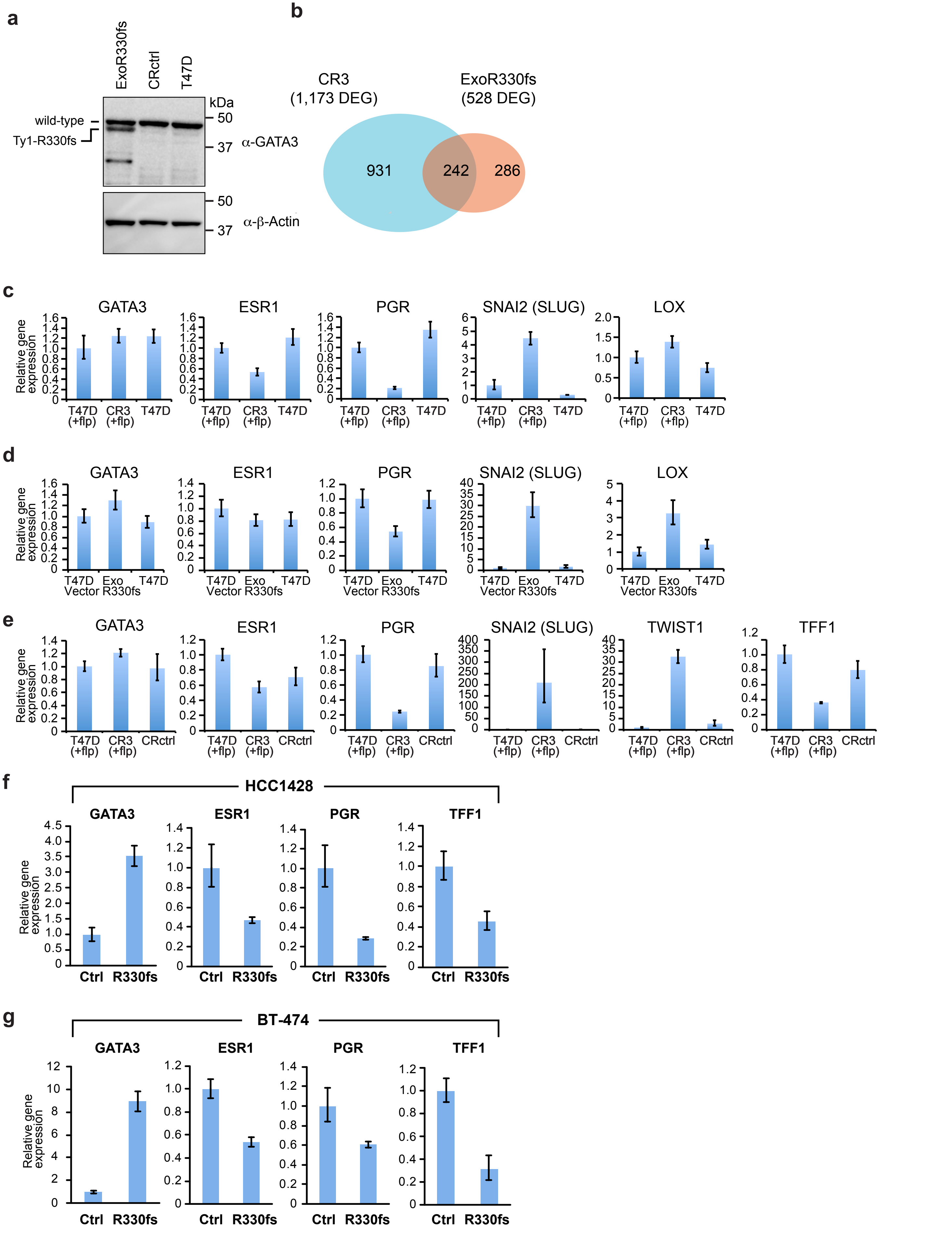
a
ExoR330fs
CRctrl
T47D
kDa
wild-type
Ty1-R330fs
50
37
α-GATA3
50
37
α-β-Actin
b
CR3
(1,173 DEG)
ExoR330fs
(528 DEG)
931
242
286
c
GATA3
ESR1
PGR
SNAI2 (SLUG)
LOX
Relative gene expression
T47D (+flp)
CR3 (+flp)
T47D
d
GATA3
ESR1
PGR
SNAI2 (SLUG)
LOX
Relative gene expression
T47D Vector
Exo R330fs
T47D
e
GATA3
ESR1
PGR
SNAI2 (SLUG)
TWIST1
TFF1
Relative gene expression
T47D (+flp)
CR3 (+flp)
CRctrl
f
HCC1428
GATA3
ESR1
PGR
TFF1
Relative gene expression
Ctrl
R330fs
g
BT-474
GATA3
ESR1
PGR
TFF1
Relative gene expression
Ctrl
R330fs

Supplementary Figure 3. R330fs mutation alters steroid hormone network genes.
(**a**) Immunoblot analysis of the ExoR330fs and control CRISPR-Cas9 clone cells (CRctrl). The CRctrl clone was generated by the same method for the CR3 clone, except wild-type GATA3 gene template was used as the donor DNA. (**b**) Venn diagram showing the overlap of differentially expressed genes between CR3 and ExoR330fs cells. (**c**) Gene expression validation by qPCR in CR3 cells. (**d**) Gene expression analysis by qPCR in ExoR330fs cells. (**e**) Gene expression analysis by qPCR in CRctrl cells. (**f**) Gene expression analysis by qPCR in HCC-1428 cells. (**g**) Gene expression analysis by qPCR in BT-474 cells.
(**c-f**) Relative gene expression was calculated by the delta-delta Ct method. The experimental data are presented as means +/- standard deviations from three biological replicates.

Takaku et al. Supplementary Figure 4

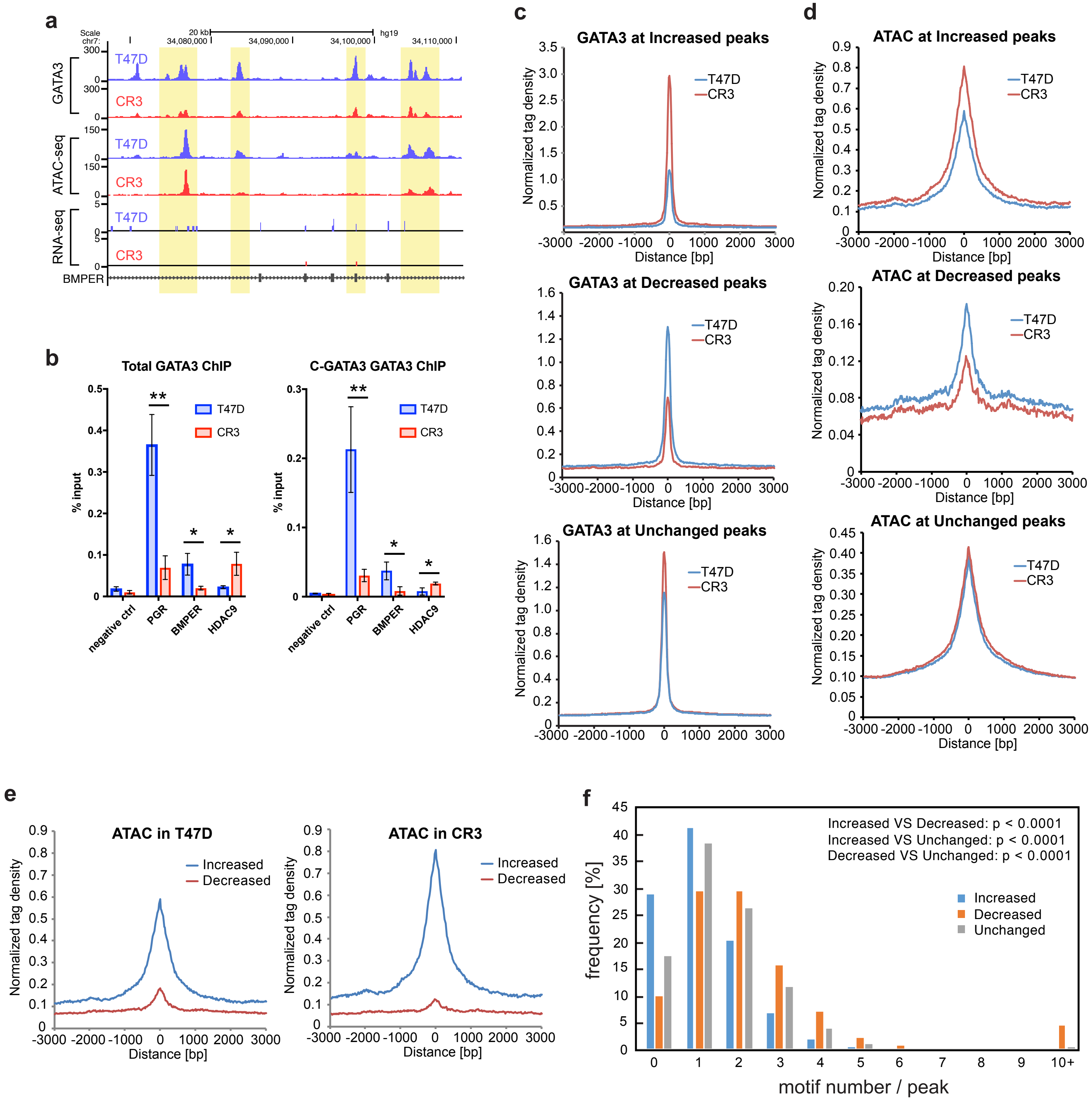

Supplementary Figure 4. Differential GATA3 binding in CR3 cells.
(**a**) Decreased GATA3 binding at the BMPER locus. UCSC Genome Browser views showing the mapped read coverage of GATA3 ChIP-seq, ATAC-seq, and RNA-seq data. (**b**) ChIP-qPCR analysis at differential binding sites. Decreased binding (shown in Fig. 4a left, Supplementary fig. 4a) and increased binding (shown in Fig.4a right) were confirmed by real-time PCR analysis. N-terminal specific GATA3 antibody (left) or C-terminal specific GATA3 antibody (right) was used (n=3). (**c-e**) Metaplots of normalized GATA3 ChIP-seq and ATAC-seq signals in each GATA3 peak groups. (**f**) Bar graphs showing the observed motif frequency in each GATA3 peak group. The number of the consensus motifs (WGATAR) within each peak (399bp) was calculated.

Takaku et al. Supplementary Figure 5

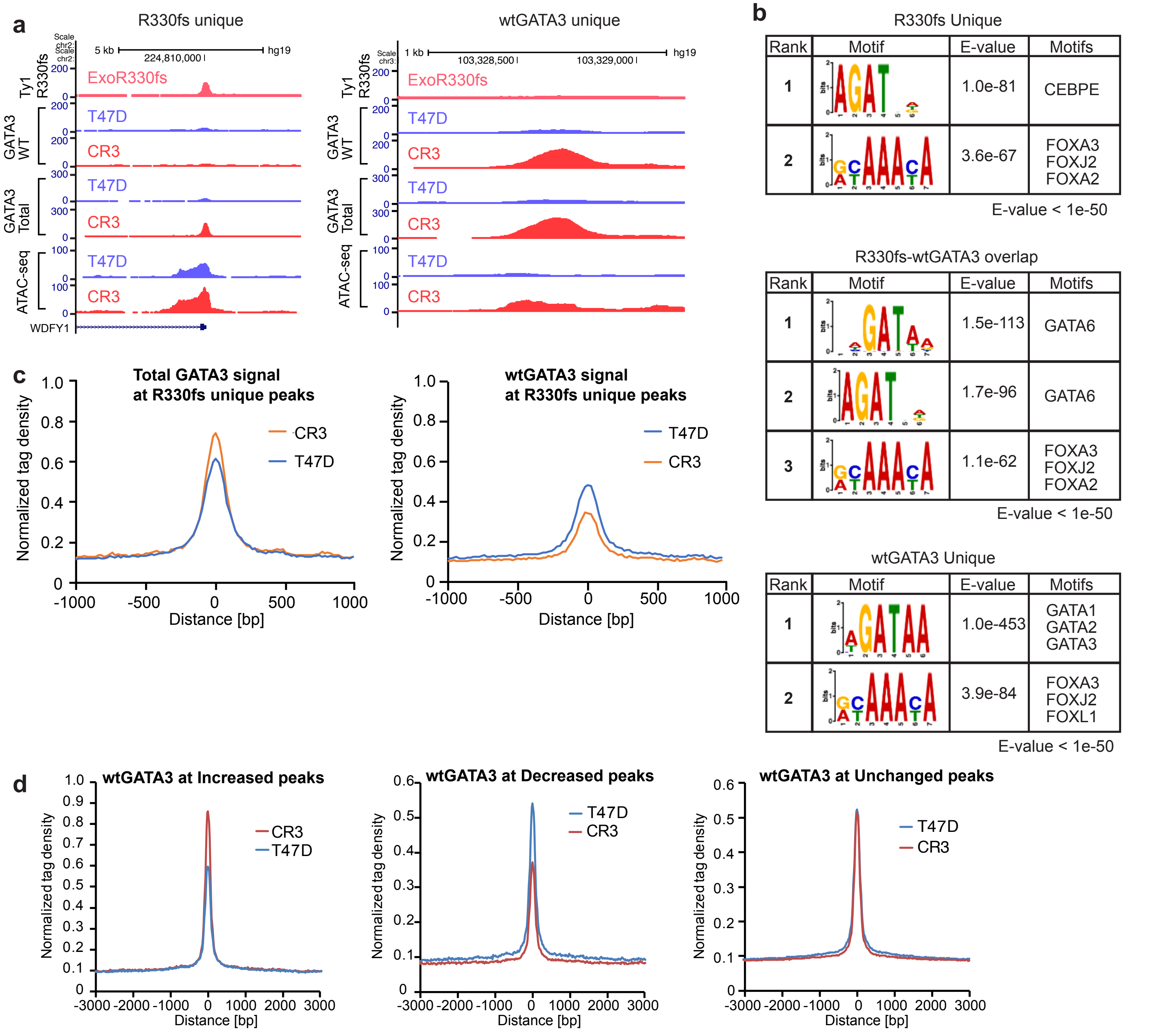

Supplementary Figure 5. R330fs mutant alters wtGATA3 DNA binding activity.
(**a**) Representative genome browser tracks at R330fs mutant or wild-type GATA3 unique binding locus. (**b**) Motif enrichment analysis in each peak group. The significantly enriched motifs (E-value < 1e-50) are represented. (**c**) Metaplots of normalized total GATA3 ChIP-seq signals (left) and wild-type GATA3 ChIP-seq signals at R330fs unique peak regions. (**d**) Metaplots of normalized wtGATA3 ChIP-seq signals in total GATA3 peak group.

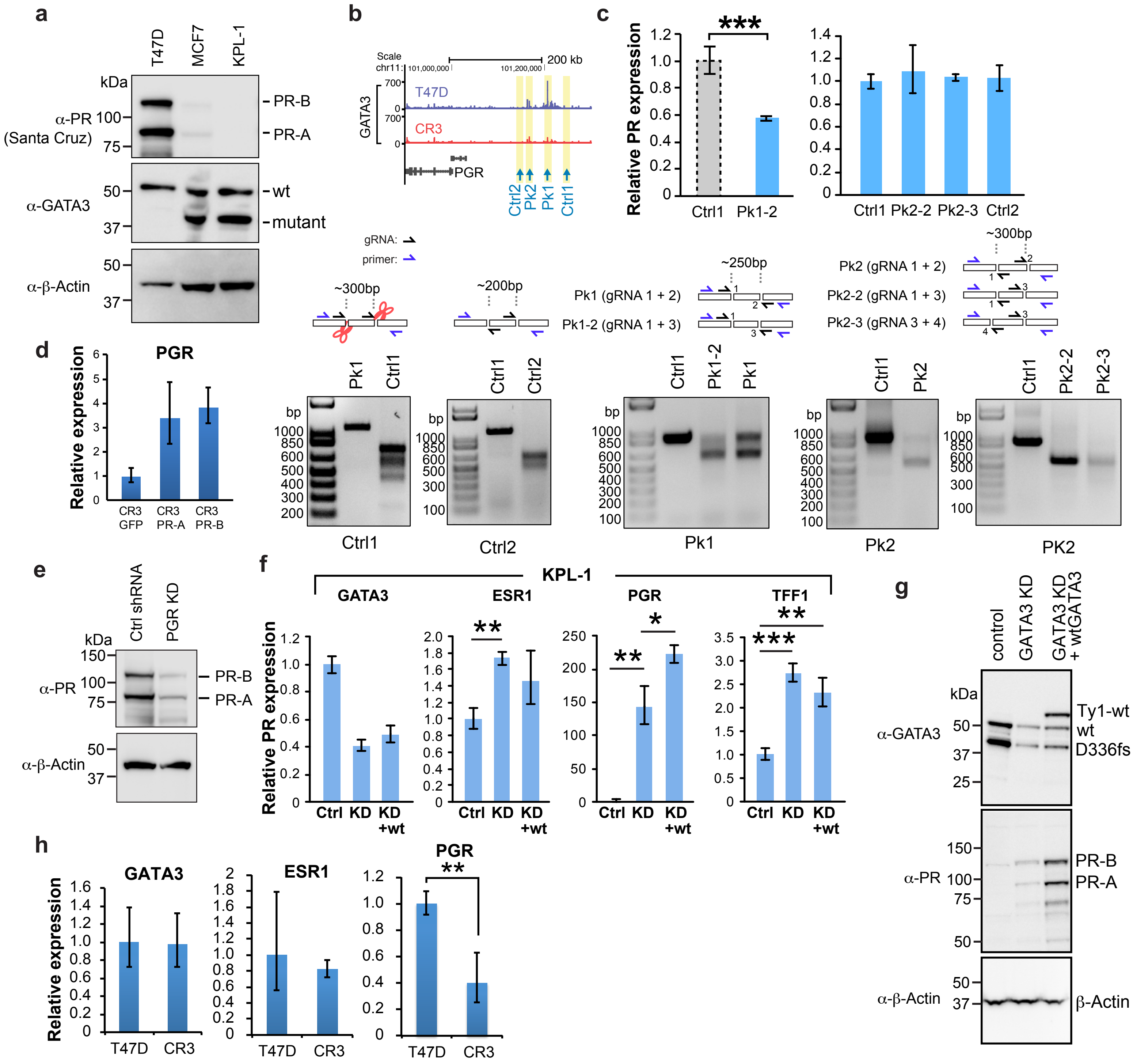

Supplementary Figure 6. ZnFn2 mutations decrease the expression level of progesterone receptor. (**a**) Immunoblot analysis of PR in GATA3 mutant breast cancer cell lines. (**b**) PCR validation of the GATA3-peak deletions by CRISPR-Cas9. For each deletion, two gRNAs (black arrows) were used. The primers (blue arrows) were designed to amplify each region. The gRNAs for Pk1 and Pk2 deletions are located at GATA3 binding sites, which includes GATA3 consensus motifs, while the gRNAs for Ctrl1 and Ctrl2 were located at the loci where GATA3 ChIP-seq signals were the background level (top genome browser view). The different combinations of gRNAs were used for validation. (**c**) PR expression analysis in the GATA3-peak depleted cells by CRISPR-Cas9. Relative expression was calculated by the delta-delta Ct method and compared to the expression level of control cells. For the Pk1-2 analysis, the same conrtol data shown in Figure 6B was used. (**d**) PR overexpression in CR3 cells. (**e**) Immunoblot analysis of PR in the PR knockdown T47D cells. (**f**) Gene expression analyis in KPL-1 cells. 'KD' indicates GATA3 knockdown, while 'wt' indicates overexpression of wild-type GATA3. (**g**) Immunoblot analysis of KPL-1 cells shown in panel f. (**h**) Gene expression in xenograft tumor samples. RNAs were isolated from the xenograft tumors (N=3). Relative gene expression was calculated by the delta-delta Ct method.

a

Upstream regulator	p-value	# genes	Predicted activation
PGR	9.20E-25	47	Activated
NR3C1	6.40E-20	65	Activated
progesterone	5.91E-18	58	Activated
TNF	1.37E-15	104	Activated
R5020	1.37E-13	19	Activated

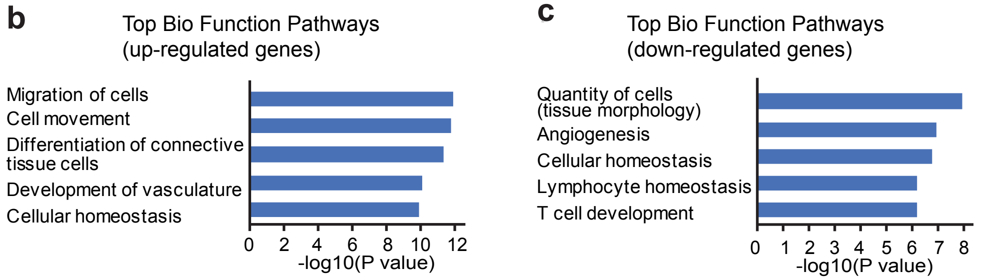

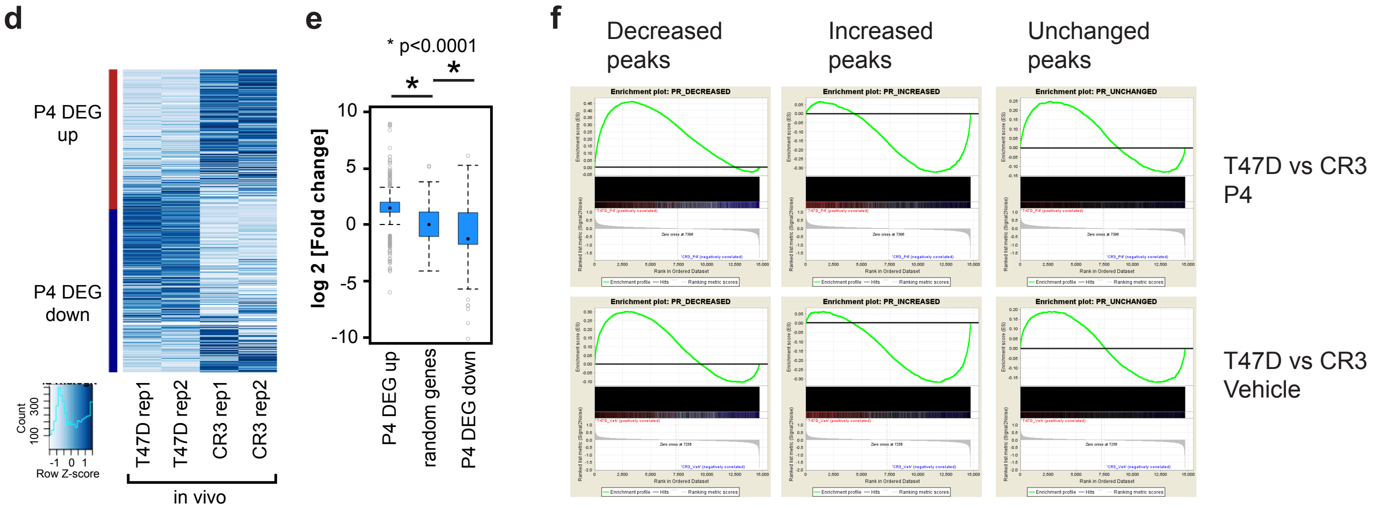

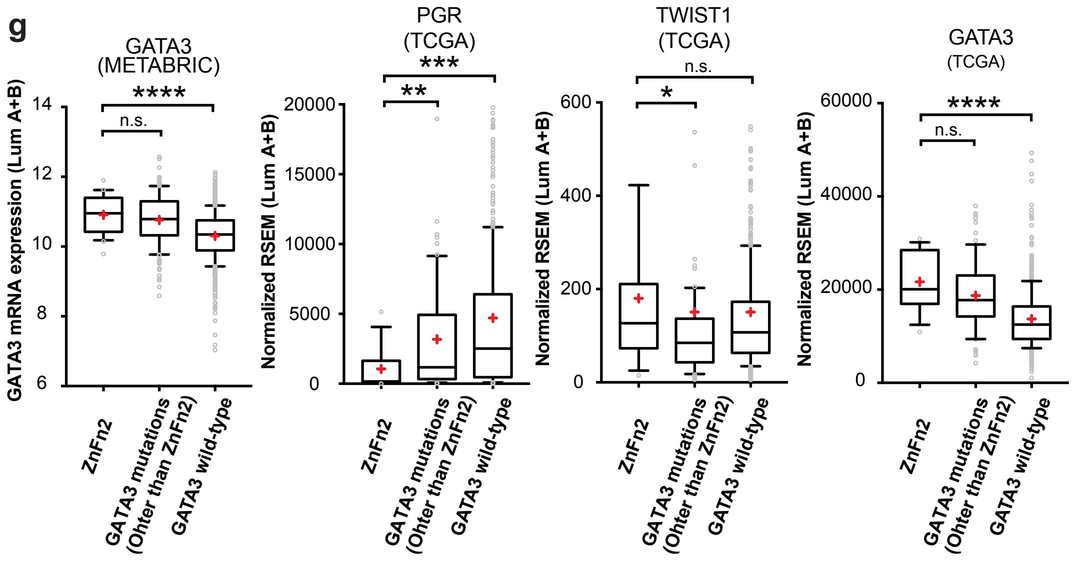

h

	PR positive	PR negative	P value (ZnFn2 vs
ZnFn2	7	9	-
Other mutations	59	8	0.0004
GATA3 WT	413	54	<0.0001

Supplementary Figure 7. PR actions are altered in ZnFn2 mutant cells.
(**a-c**) IPA analysis of DEG in T47D cells by progesterone treatment. (**a**) The upstream regulators of up-regulated genes by progesterone treatment are indicated. (**b-c**) The predicted top 5 Bio Function Pathways are indicated. (**d**)Heatmap showing the row-scaled FPKM values of the in vivo RNA-seq data. The differentially expressed genes between progesterone treated CR3 cells v.s. T47D control cells (identified by the in vitro RNA-seq shown in Fig. 7a and Supplementary table 1) are categorized into up- and down- regulated gene groups. (**e**) Box-and-whisker plots showing relative fold changes in each category. Random genes indicates the randomly selected subset of 600 genes that were not categorized as up- or down- regulated genes. (**f**) GSEA analysis showing the correlation between each class of PR binding and gene expression with (top) or without (bottom) progesterone stimulation. (**g**) GATA3, PR and TWIST1 expression in the breast cancer patients. The expression data were collected from the METABRIC or TCGA cohort. **P < 0.01, ***P < 0.001, ****P <0.0001, n.s. > 0.05 (not statistically significant), Mann-Whitney test. (**h**) PR status in ZnFn2 mutant tumors. Immunohistochemistry results are obtained from TCGA data cohort, and Fisher's exact tests were applied to test statistical differences in subtype frequencies. Because the diagnosis year in this study ranged from 1988 to 2011, the thresholds for PR positive status are potentially nonuniform (The Cancer Genome Atlas Network, 2012).

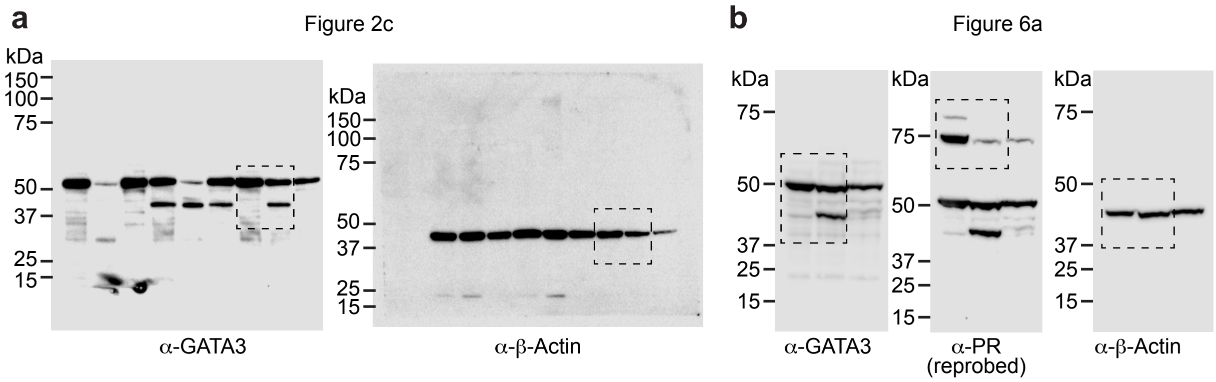

Supplementary Figure 8. Uncropped blot images.
(**a**) Uncropped immunoblot images from Figure 2c. (**b**) Uncropped immunoblot images from Figure 6a.

Supplementary Table 1. Oncomine concept analysis of up-regulated genes in CR3 cells

Concept Name	Genes	P-Value	Q-Value	Odds Ratio
**Cancer Histology**				
Ductal Breast Carcinoma Type Epithelia: Invasive Ductal Breast Carcinoma - Top 10% Over-expressed (Schuetz Breast 2)	124	1.71E-20	4.41E-18	2.9
Breast Adenocarcinoma Type: Lobular Breast Carcinoma - Top 10% Over-expressed (TCGA Breast)	110	2.39E-13	2.80E-11	2.4
Breast Adenocarcinoma Type: Lobular Breast Carcinoma - Top 10% Over-expressed (Zhao Breast)	70	2.80E-07	1.14E-05	2.1
**Clinical outcome**				
Breast Carcinoma - Metastatic Event at 3 Years - Top 10% Over-expressed (Kao Breast)	95	3.41E-09	2.04E-07	2.1
**Drug Sensitivity**				
Erlotinib Sensitive - Breast Cancer Cell Line - Top 10% Over-expressed (Hoeflich CellLine 2)	142	1.78E-30	1.14E-27	3.6
Paclitaxel Sensitive - Multi-cancer Cell Line - Top 10% Over-expressed (Garnett CellLine)	119	1.36E-29	8.16E-27	4.2
CGP-60474 Sensitive - Multi-cancer Cell Line - Top 10% Over-expressed (Garnett CellLine)	118	3.90E-29	2.22E-26	4.1
**Biomarker analysis**				
Breast Cancer Cell Line - EGFR Western Blot Protein Level - Positive - Top 10% Over-expressed (Hoeflich CellLine 2)	122	1.59E-19	3.69E-17	2.9
Ductal Breast Carcinoma - ERBB2 Positive - Top 10% Over-expressed (Esserman Breast)	106	1.92E-12	1.97E-10	2.3
Invasive Breast Carcinoma - ERBB2 Positive - Top 5% Over-expressed (Stickeler Breast)	62	4.07E-10	2.90E-08	2.6
**Pathology subtype grade**				
Ductal Breast Carcinoma in Situ Stroma - High Grade - Top 5% Over-expressed (Ma Breast 4)	76	3.90E-17	6.95E-15	3.4
Ductal Breast Carcinoma - High Grade - Top 1% Over-expressed (Perou Breast)	11	2.51E-05	6.19E-04	5.4
**Pathology subtype Stage**				
Breast Carcinoma - Advanced N Stage - Top 10% Over-expressed (Loi Breast)	97	5.02E-10	3.52E-08	2.2
Breast Carcinoma - Advanced N Stage - Top 10% Over-expressed (Yu Breast 3)	61	7.92E-09	4.43E-07	2.5

The up-regulated genes in CR3 cells were applied to the Oncomine concept analysis. The significanly associated clinical data sets (Odds > 2, P-value < 1E-4) are represented. Size: number of genes both concepts share in common, Q-value: estimated false discovery rates.

Supplementary Table 2. Oncomine concept analysis of down-regulated genes in CR3 cells

Concept Name	Size	P-Value	Q-Value	Odds Ratio
**Cancer Histology**				
Breast Adenocarcinoma Type: Medullary Breast Carcinoma - Top 5% Under-expressed (Curtis Breast)	72	8.14E-16	9.40E-13	3.3
Breast Adenocarcinoma Type: Medullary Breast Carcinoma - Top 10% Under-expressed (Ginestier Breast)	100	1.61E-12	8.53E-10	2.4
Breast Adenocarcinoma Type: Ductal Breast Carcinoma - Top 10% Under-expressed (Lu Breast)	91	5.78E-09	1.19E-06	2.1
**Clinical outcome**				
Ductal Breast Carcinoma - Recurrence at 1 Year - Top 5% Under-expressed (Esserman Breast)	69	3.49E-15	3.26E-12	3.3
Ductal Breast Carcinoma Epithelia - Dead at 1 Year - Top 5% Under-expressed (Boersma Breast)	48	1.84E-10	6.08E-08	3.2
Breast Carcinoma - Recurrence at 5 Years - Top 10% Under-expressed (vandeVijver Breast)	79	7.95E-09	1.53E-06	2.2
**Drug Sensitivity**				
Nilotinib Sensitive - Multi-cancer Cell Line - Top 5% Under-expressed (Barretina CellLine)	70	2.11E-15	2.09E-12	3.3
PLX4720 Sensitive - Multi-cancer Cell Line - Top 5% Under-expressed (Garnett CellLine)	50	1.49E-11	6.51E-09	3.4
CHIR-265 Sensitive - Multi-cancer Cell Line - Top 10% Under-expressed (Barretina CellLine)	92	9.15E-10	2.46E-07	2.2
**Biomarker analysis**				
Breast Carcinoma - ERBB2/ER/PR Negative - Top 5% Under-expressed (Waddell Breast)	71	3.85E-17	5.90E-14	3.6
Ductal Breast Carcinoma - ERBB2/ER/PR Negative - Top 10% Under-expressed (Bittner Breast)	108	1.01E-15	1.11E-12	2.7
Ductal Breast Carcinoma - ERBB2/ER/PR Negative - Top 5% Under-expressed (Esserman Breast)	67	1.06E-13	7.56E-11	3.1
**Pathology subtype grade**				
Ductal Breast Carcinoma - High Grade - Top 10% Under-expressed (Bittner Breast)	111	1.04E-16	1.46E-13	2.7
Invasive Breast Carcinoma - High Grade - Top 10% Under-expressed (Gluck Breast)	91	3.45E-10	1.07E-07	2.2
Ductal Breast Carcinoma - High Grade - Top 10% Under-expressed (Esserman Breast)	92	8.75E-10	2.38E-07	2.2
**Pathology subtype Stage**				
Breast Carcinoma - Advanced N Stage - Top 5% Under-expressed (Miyake Breast)	62	5.39E-12	2.67E-09	3

The down-regulated genes in CR3 cells were applied to the Oncomine concept analysis. The significanly associated clinical data sets (Odds > 2, P-value < 1E-4) are represented. Size: number of genes both concepts share in common, Q-value: estimated false discovery rates.

Supplementary Table 3. Oncomine concept analysis of uniquely upregulated genes in progesterone treated T47D cells

Concept Name	Size	P-Value	Q-Value	Odds Ratio
**Cancer Histology**				
Breast Adenocarcinoma Type: Medullary Breast Carcinoma - Top 10% Under-expressed (Ginestier Breast)	68	1.35E-07	4.36E-05	2.2
Breast Adenocarcinoma Type: Medullary Breast Carcinoma - Top 5% Under-expressed (Curtis Breast)	38	1.10E-05	0.001	2.3
**Clinical outcome**				
Breast Carcinoma - Dead at 3 Years - Top 5% Under-expressed (Pawitan Breast)	38	4.52E-06	6.65E-04	2.4
Breast Carcinoma - Metastatic Event at 5 Years - Top 5% Under-expressed (vantVeer Breast)	27	8.12E-05	0.006	2.4
Breast Carcinoma - Recurrence at 5 Years - Top 10% Under-expressed (vandeVijver Breast)	79	7.95E-09	1.53E-06	2.2
**Pathology subtype grade**				
Lobular Breast Carcinoma - High Bloom-Richardson Grade - Top 10% Under-expressed (Lu Breast)	67	2.95E-07	7.99E-05	2.1
Ductal Breast Carcinoma - High Grade - Top 5% Under-expressed (Bonnefoi Breast)	40	2.24E-06	3.84E-04	2.4
Breast Carcinoma - High Elston Grade - Top 10% Under-expressed (Ivshina Breast)	49	2.10E-05	0.002	2.1
**Pathology subtype Stage**				
Breast Carcinoma - Advanced M Stage - Top 5% Under-expressed (vantVeer Breast)	27	8.12E-05	0.006	2.4

427 uniquely up-regulated genes in progesterone-treated T47D cells were applied to the Oncomine concept analysis. The significanly associated clinical data sets (Odds > 2, P-value < 1E-4) are represented. Size: number of genes both concepts share in common, Q-value: estimated false discovery rates.

Supplementary Table 4. Primer sequences

Oligo Name	Sequence
CRISPR gRNA GATA3 F1	CACCG ACAACCCCCTTGGTATGCAT
CRISPR gRNA GATA3 R1	AAAC ATGCATACCAAGGGGGTTGT C
CRISPR gRNA PGR Pk1 F1	CACCG CATGAAAACCATTAAGGGTC
CRISPR gRNA PGR Pk1 R1	AAAC GACCCTTAATGGTTTTCATG C
CRISPR gRNA PGR Pk1 F2	CACCG TGAGTTCTTTTCCGTCATGA
CRISPR gRNA PGR Pk1 R2	AAAC TCATGACGGAAAAGAACTCA C
CRISPR gRNA PGR Pk1 F3	CACCG GTGAGTTCTTTTCCGTCATG
CRISPR gRNA PGR Pk1 R3	AAAC CATGACGGAAAAGAACTCAC C
CRISPR gRNA PGR Pk2 F1	CACCG GGGGGAGAGTTCTATGTCAA
CRISPR gRNA PGR Pk2 R1	AAAC TTGACATAGAACTCTCCCCC C
CRISPR gRNA PGR Pk2 F2	CACCG GTATCAGCTCTGGTAGTATA
CRISPR gRNA PGR Pk2 R2	AAAC TATACTACCAGAGCTGATAC C
CRISPR gRNA PGR Pk2 F3	CACCG AGTATCAGCTCTGGTAGTAT
CRISPR gRNA PGR Pk2 R3	AAAC ATACTACCAGAGCTGATACT C
CRISPR gRNA PGR Pk2 F4	CACCG ATGTCAAGGGAACACCCTGT
CRISPR gRNA PGR Pk2 R4	AAAC ACAGGGTGTTCCCTTGACAT C
CRISPR gRNA PGR ctrl1 F1	CACCG GAAGTACCCCTATATCGTCA
CRISPR gRNA PGR ctrl1 R1	AAAC TGACGATATAGGGGTACTTC C
CRISPR gRNA PGR ctrl1 F2	CACCG CATCTGAAGGCTTAGATCAA
CRISPR gRNA PGR ctrl1 R2	AAAC TTGATCTAAGCCTTCAGATG C
CRISPR gRNA PGR ctrl2 F1	CACCG TACCAGTTTAGCCCGTTACC
CRISPR gRNA PGR ctrl2 R1	AAAC GGTAACGGGCTAAACTGGTA C
CRISPR gRNA PGR ctrl2 F2	CACCG GAGAAGTCCTGCATTGCATC
CRISPR gRNA PGR ctrl2 R2	AAAC GATGCAATGCAGGACTTCTC C
PGR_Enh_Pk1 confirm F1	TGTTCCTTCAAAGGATTAGTTTAACAT
PGR_Enh_Pk1 confirm R1	ACTCAATGGAGTAATACTGGTTCTGTT
PGR_Enh_Pk2 confirm F1	TCTTTATTTCCTGAAGTTTTGATTGAT
PGR_Enh_Pk2 confirm R1	ATAATTCTCCTGGGAACATAACTCC
PGR_Enh_Ctrl1 confirm F1	TGGAGAAAAGACCAACTCTAGAAATAA
PGR_Enh_Ctrl1 confirm R1	TAACTTTGCCTGCTATACTCTGAAAAT
PGR_Enh_Ctrl2 confirm F1	GATTCTCCTCTTAGAATGGAAGCTTAT
PGR_Enh_Ctrl2 confirm R1	GTACAAAATTGTGGGTGTCACATAATA
Donor template F	CTTTGCTGTGAATGCATCCTGATGGCTGGA
Donor template R	CACTGCACCAGGGCCACAGCGAGAGAGTTG
HDAC9_ChIP_F	CGAGGTACTCCCAGAAGCAG
HDAC9_ChIP_R	GCAAATCATCCAGGAAAACC
PGR_ChIP_F	AGCAGCATTCCCAGAAAAGA
PGR_ChIP_R	TACACAAGGGTGTCCTGACC
PGR_ChIP_F	TGAGCTGCAACTGTGTTCCT
PGR_ChIP_R	TTGCAGTATTGTGGCAAAGC
Negative_Control_ChIP_F	TGCGGTACATGCACTTCAAT
Negative_Control_ChIP_R	GGCAACCATTGTCCTGACTT
CR3_confirm_F	CTCTCTCCCCACTCTCAGTCTGCAG
CR3_confirm_R	GCTGATCCCAGTCCACTTACATTGTGAAG

The primers used in this study are listed.

Supplementary Table 5. Antibody information

Data Type	antibody	input DNA	Kit	PCR cycles	Replicates	Normalized counts: Heatmap	Normalized counts: Genome Browser	GEO
GATA3 ChIP-seq	Cell Signaling, D13C9	2 ng	NEXTflex Rapid DNA-Seq Kit - BIOO SCIENTIFIC	12	2	35,000,000	43,322,736	GSE99479
PR ChIP-seq	Santa Cruz, H-190	1 ng	NEXTflex Rapid DNA-Seq Kit - BIOO SCIENTIFIC	12	2	50,000,000	40,137,906	GSE99479
Ty-1 (R330fs) ChIP-seq	Menafra et al., 2014	<1 ng	NEXTflex Rapid DNA-Seq Kit - BIOO SCIENTIFIC	12	2	-	45,001,539	GSE99479
GATA3 (C-terminus) ChIP-seq	ThermoFisher, PA5-20892	2 ng	NEXTflex Rapid DNA-Seq Kit - BIOO SCIENTIFIC	12	2	-	46,249,871	GSE99479
ATAC-seq	N/A	25,000 cells	Nextera DNA Library Preparation Kit - Illumina	8	3	-	43,869,357	GSE99479
PR Western blot (Figure 6)	Cell Signaling, C89F7, x1,000 dilution							
β-actin Western blot	abcam, ab8226, x1,000 dilution							

The information of the antibodies used in this study is listed.
