# Supplementary material for: GATA3 zinc finger 2 mutations reprogram the breast cancer transcriptional network
Source: Nat Commun. 2018 Mar 13;9:1059. doi: 10.1038/s41467-018-03478-4 (PMC5849768; doi:10.1038/s41467-018-03478-4)
Supplement: Supplementary file 2 — Description of Additional Supplementary Files [file 41467_2018_3478_MOESM2_ESM.pdf]

Description of Additional Supplementary Files

File Name: Supplementary Data 1
Description: Differentially expressed genes
